# Physiological and transcriptome analysis of He-Ne laser pretreated wheat seedlings in response to drought stress

**DOI:** 10.1038/s41598-017-06518-z

**Published:** 2017-07-21

**Authors:** Zongbo Qiu, Mengmeng Yuan, Yanyan He, Yongfang Li, Liang Zhang

**Affiliations:** College of Life Science, Henan Normal University, Xinxiang, 453007 P.R. China

## Abstract

Drought stress is a serious problem worldwide that reduces crop productivity. The laser has been shown to play a positive physiological role in enhancing plant seedlings tolerance to various abiotic stresses. However, little information is available about the molecular mechanism of He-Ne laser irradiation induced physiological changes for wheat adapting to drought conditions. Here, we performed a large-scale transcriptome sequencing to determine the molecular roles of He-Ne laser pretreated wheat seedlings under drought stress. There were 98.822 transcripts identified, and, among them, 820 transcripts were found to be differentially expressed in He-Ne laser pretreated wheat seedlings under drought stress compared with drought stress alone. Furthermore, most representative transcripts related to photosynthesis, nutrient uptake and transport, homeostasis control of reactive oxygen species and transcriptional regulation were expressed predominantly in He-Ne laser pretreated wheat seedlings. Thus, the up-regulated physiological processes of photosynthesis, antioxidation and osmotic accumulation because of the modified expressions of the related genes could contribute to the enhanced drought tolerance induced by He-Ne laser pretreatment. These findings will expand our understanding of the complex molecular events associated with drought tolerance conferred by laser irradiation in wheat and provide abundant genetic resources for future studies on plant adaptability to environmental stresses.

## Introduction

Wheat is one of the most important staple crops in the world by virtue of its key contribution to food security. Drought stress severely limits crop production and reduces the yield and quality of wheat^1^. Importantly, drought stress is predicted to become more frequent and severe due to the future climate change, and thus poses a serious challenge to agricultural production worldwide^1, 2^. Therefore, understanding the molecular mechanisms responsible for plant drought stress tolerance is essential for improving this beneficial trait in crops.

With the development of laser technology, laser has been widely used in the field of biology. Low dose of laser irradiation can promote plant growth and physiological metabolism^3–5^ and also protect plant seedlings against enhanced UV-B radiation^6^, chilling stress^7^, osmotic stress^8^, drought stress^9^, and cadmium stress^10^. Laser irradiation enhanced plants tolerance mainly through improving seeds germination rates, plant height, root length, seedlings biomass, photosynthesis, better maintenance of reactive oxygen species (ROS) homeostasis and membrane stability at the physiological level in crops^11, 12^. Gao *et al*. have illustrated that He-Ne alleviated the adverse impacts of salt stress on plant growth by enhancing relative water content, and meanwhile, increasing chlorophyll concentrations and the activity of photosystem II resulting in improvement of plant photosynthetic activity^11^. Our previous investigations showed that He-Ne and CO_2_ laser pretreatment could enhance drought tolerance of wheat seedlings by increasing the concentration of ascorbic acid (AsA) and glutathione (GSH) and the activities of enzymes involved in reactive oxygen species (ROS) scavenging including superoxide dismutase (SOD), peroxidase (POD), catalase (CAT), ascorbate peroxidase (APX)^8, 9^. Although much effort has focused on elucidating the mechanism of action of laser irradiation at morphological and physiological levels, such as seed germination, photosynthetic rate, chlorophyll concentration, antioxidant compounds production and antioxidant enzymatic activities^6, 11–13^, the molecular basis of He-Ne laser irradiation induced physiological changes for crops adapting to drought environments is still poorly understood due to the absence of genomic information.

A rapid and cost-effective approach to explore plant tolerance mechanisms to abiotic stresses is to carry out transcriptomic analyses using microarray-based or RNA-Seq-based technologies. More recently, transcriptome sequencing has proven to be a powerful tool to discover the molecular processes involved in plant response to various abiotic stresses. Genome-scale transcriptome analyses have been employed on diverse crops based on Illumina RNA-Seq to monitor gene expression in response to heat stress^14^, cold stress^15^ and drought stress^16–18^. A recent genome-wide identification of differentially expressed transcript derived fragments from water deficit stressed root and leaf tissues in tetraploid cotton provided their gene ontology, functional/biological distribution, and possible roles of gene duplication^19^. Furthermore, Fracasso *et al*. compared drought response of two sorghum genotypes characterized by contrasting water use efficiency using high-throughput sequencing^2^. Although much progress has been made in exploring the adaptive mechanisms of plants to drought stress, genome-wide transcriptomic analysis of drought stress tolerance conferred by laser irradiation in wheat has not been reported. Using the high-throughput sequencing, we studied He-Ne laser irradiation induced transcriptome changes in wheat seedlings under drought stress, and believe it is important for understanding of the molecular mechanism associated with laser irradiation mediated drought tolerance in wheat.

## Materials and Methods

### Plant material and treatment conditions

Seeds of wheat (Triticum *aestivum* L. cv. Aikang No. 58, obtained from Henan Academy of Agricultural Sciences) were surface sterilized for 3 min by immersion in 0.01% HgCl_2_, soaked in distilled water for 24 h prior to laser treatment, and then were air dried. A He-Ne laser (wavelength 632.8 nm, power density 5.43 mW mm^−2^, beam diameter 12 mm) directly irradiated the embryo of wheat seeds for 3 min. Three replications of 10 pure seeds were used for each of the different treatment. One seed was pretreated only once by He-Ne laser irradiation. The seeds were exposed to He-Ne laser one by one. The He-Ne laser (Model No. MSHN5-A-B450MM) was made at Northwest University (China). After He-Ne laser pretreatment, seeds were sown in plastic pots (8 cm diameter at top, 6 cm diameter at bottom, and 8.0 cm height) filled with a mixture of vermiculite and nutrient soil in 1:1 ratio and irrigated with tap water. After germination, the seedlings were cultured in a greenhouse under a 12 h photoperiod, 25/18 °C day/night temperatures, light intensity of 400 μmol m^−2^ s^−1^, and relative humidities of 60/75% (day/night). One-week-old seedlings (with one fully expanded leaf) were randomly divided into four groups: seedlings with no drought stress and no He-Ne laser irradiation were regarded as the control group (CK), seedlings treated with natural drought stress without water for 5 days (the soil relative water content was 50%, P), seedlings pretreated with He-Ne laser irradiation alone (L), seedlings pretreated with He-Ne laser irradiation before treated with natural drought stress without water for 5 days (L + P). On the 5th day of drought stress, leaves and roots were collected, respectively, and immediately frozen in liquid nitrogen and stored at −80 °C until further use.

### Growth parameters

Growth parameters (plant height, root length, shoot dry weight and root dry weight) were determined 5 days after He-Ne laser pretreatment or drought stress. Relative water content (RWC) in leaves was calculated according to the following formula^20^:

$${\rm{RWC}}( \% )=[({\rm{fresh}}\,{\rm{weight}}-{\rm{dry}}\,{\rm{weight}})/({\rm{turgidweight}}-{\rm{dry}}\,{\rm{weight}})]\times 100$$

Leaves of the different treated seedlings were collected and immediately weighed (fresh weight, FW). They were rehydrated in water for 24 h until fully turgid, surface dried, and reweighed (turgid weight, TW). The tissues were then oven dried at 105 °C for 24 h and re-weighed (dry weight, DW).

### Determination of malondialdehyde (MDA), photosynthetic pigments and protein concentration

Lipid peroxidation was assayed by measuring malondialdehyde (MDA) content using the method described by Qiu *et al*.^21^. The content of chlorophyll *a*, chlorophyll *b*, and total chlorophyll in wheat seedling leaves were determined spectrophotometrically according to the method of Lichtenthaler^22^. Soluble protein content in wheat seedlings leaves was carried out according to the method described by Bradford using bovine serum albumin as a standard^23^.

### Total RNA extraction, libraries construction and illumina sequencing

Twenty individual wheat seedlings were pooled to create one treatment and total RNA was extracted from different treatment using RNAsimple total RNA kit (Tiangen, China) according to the manufacturer’s instructions. The concentration and quality of total RNA in each sample was determined using an Agilent 2100 Bioanalyzer (Agilent Technologies, Santa Clara, CA, USA). The library was constructed using the NEB Next Ultra Directional RNA Library Prep Kit for Illumina (NEB, Ispawich, USA) following manufacturer’s instructions and four index codes were added to attribute sequences to different samples. Briefly, mRNAs were enriched from 3 ug total RNA of wheat tissues using magnetic beads with Oligo (dT) (Life technologies, CA, USA), and then fragmented using divalent cations under elevated temperature in the NEB proprietary fragmentation buffer. Using these short fragments as templates, a random hexamer primer was used to synthesize first-strand cDNA. Second-stranded cDNAs were then synthesized using RNase H and DNA polymerase I. These cDNA fragments were ligated with the adapters, and these products were then purified and enriched with PCR to create the final cDNA libraries. Finally, the library was sequenced by Novogene Bioinformatics Technology Co., Ltd (Beijing, China) on an Illumina HiSeq^TM^ 2000 platform. The clean reads were first obtained by removing low-quality reads and reads containing adapters or poly-N stretches from the raw data. Then, clean reads were mapped to our assembled wheat transcriptome (SRR5119530) by using TopHat software (v2.0.12).

To maximize the number of genes included in wheat transcriptome, a cDNA sample was prepared from an equal mixture of total RNA isolated from different treatment wheat seedlings (with one fully expanded leaf), and sequenced using the Illumina high-throughput sequencing platform. The different treatment wheat seedlings were divided into four groups: seedlings with no drought stress and no He-Ne laser irradiation, seedlings treated with natural drought stress, seedlings were pretreated with He-Ne laser irradiation alone, and seedlings pretreated with He-Ne laser irradiation before treated with natural drought stress. Raw sequence data are available in the NCBI’s Sequence Read Archive (SRA) database with accession number SRR5119530. Generated clean reads were aligned to the IWGSC wheat assembly (http://archive.plants.ensembl.org/) and TGACv1 wheat assembly (http://plants.ensembl.org/) using BWA and Bowtie2 software^24^. The assembled wheat transcriptome contains 188,334 transcripts with a mean length of 801 bp and 119,588 unigene with a mean length of 1,096 bp. The summary for the wheat transcriptome is shown in Tables S1 and S2.

### Differentially expressed genes (DEGs) detection and functional analysis of DEGs

For gene expression analysis, the read numbers mapped to each gene were counted using HTSeq v0.5.4p3 and then normalized to RPKM^25^ (reads per kilobase per million mapped reads). Differential expression analysis for each sample was performed using the DESeq R package. We use false discovery rate (FDR) ≤ 0.05 and the absolute value of log2 (ratio) ≥ 1 as the threshold for judging the significance of differentially expressed gene in different treatments. Gene Ontology (GO) enrichment analysis of differentially expressed genes was implemented using the GOseq R package and GO terms with *q* < 0.05 were regarded as significantly enriched. Kyoto Encyclopedia of Genes and Genomes (KEGG) pathways analysis of the differential expression genes was performed using the KOBAS software.

### Quantitative real-time PCR

To validate the RNA-seq data, 9 genes were randomly selected to be analyzed by qRT-PCR with a reference gene (Tubulin; Table S3) and specific primer pairs for selected genes were designed as shown in Table S3. Sample treatment and RNA isolation were obtained following previously described above. The reverse-transcription reactions were performed using the iScriptTM advanced cDNA Synthesis Kit (Promega, WI, USA). Quantitative real time PCR was performed using a Rotor-Gene 3000 real-time PCR detection system (Qiagen) with SYBR^®^ qPCR Mix (Toyobo, Tokyo, Japan) and PCR amplifications were carried out in 20 µl total volume reactions containing 2 µl diluted cDNA, 300 nM of each primer, and 10 µl of the Thunderbird SYBR Green PCR Master Mix with the following cycling conditions: 95 °C for 2 min, 35 cycles at 95 °C for 15 s, 60 °C for 15 s, and 72 °C for 20 s. Dissociation curve analysis of amplification products was performed at the end of each PCR to confirm that only one PCR product was amplified and detected. The experiment was performed with at least three independent replicates, and the comparative CT method (2^−ΔΔCt^ method) was used to analyze the expression level of the different genes^26^.

### Statistical analysis

The experiment was a completely random design with three replications, and the data presented are means ± SEs. Each independent experiment was a pooled sample from at least 20 wheat seedlings. Bars with different letters above the columns in the figures indicate significant differences at *p* < 0.05 by Duncan’s multiple range test.

## Results

### Effect of He-Ne laser pretreatment on growth parameters of wheat seedlings under drought stress

Exposure to drought stress produced a significant growth inhibition in wheat seedlings, as observed in plant height, root length, shoot dry weight and root dry weight. Compared to the control (CK), drought stress (P) caused a significant decrease (p < 0.05) in the plant height, root length, shoot dry weight and root dry weight by 16.4, 19.19, 13.3 and 16.7 percent, respectively, in wheat seedlings. In contrast, He-Ne laser pretreatment combined with drought stress (L + P) caused an increase (p < 0.05) in the plant height, root length, shoot dry weight and root dry weight by 21.8, 34.5, 11.5 and 25 percent, respectively, compared with the seedlings treated with drought stress alone (Table 1). Treatments with He-Ne laser alone (L) have little effect on plant height, root length, shoot dry weight and root dry weight in wheat seedlings compared with the control. These results suggested that He-Ne laser pretreatment was responsible for the enhancement of adaptive responses of wheat seedlings against drought stress.

**Table 1:** Effect of He-Ne laser pretreatment on root length, plant height, shoot dry weight, and root dry weight of the 7-day-old wheat seedlings treated with natural drought stress without water for 5 days.

	CK	P	L	L + P
Plant height (cm)	13.49 ± 1.34a	11.28 ± 1.31b	14.54 ± 1.01a	13.74 ± 1.01a
Root length (cm)	12.66 ± 2.31a	10.23 ± 1.89b	13.47 ± 1.85a	13.76 ± 1.10a
Shoot dry weight (g)	0.060 ± 0.005a	0.052 ± 0.003b	0.057 ± 0.003a	0.058 ± 0.004a
Root dry weight (g)	0.024 ± 0.003a	0.020 ± 0.004b	0.024 ± 0.002a	0.025 ± 0.002a

Different treatment represents: The control (CK), natural drought stress (P), 3 min laser radiation (L), 3 min laser radiation + natural drought stress (L + P). Data are means of 30 seedlings, and each mean in a line followed by a different letter indicates that there are significant differences at 0.05 level according to Duncan’s multiple range test.

### Effect of He-Ne laser pretreatment on drought stress tolerance in wheat seedlings

**Figure 1:**
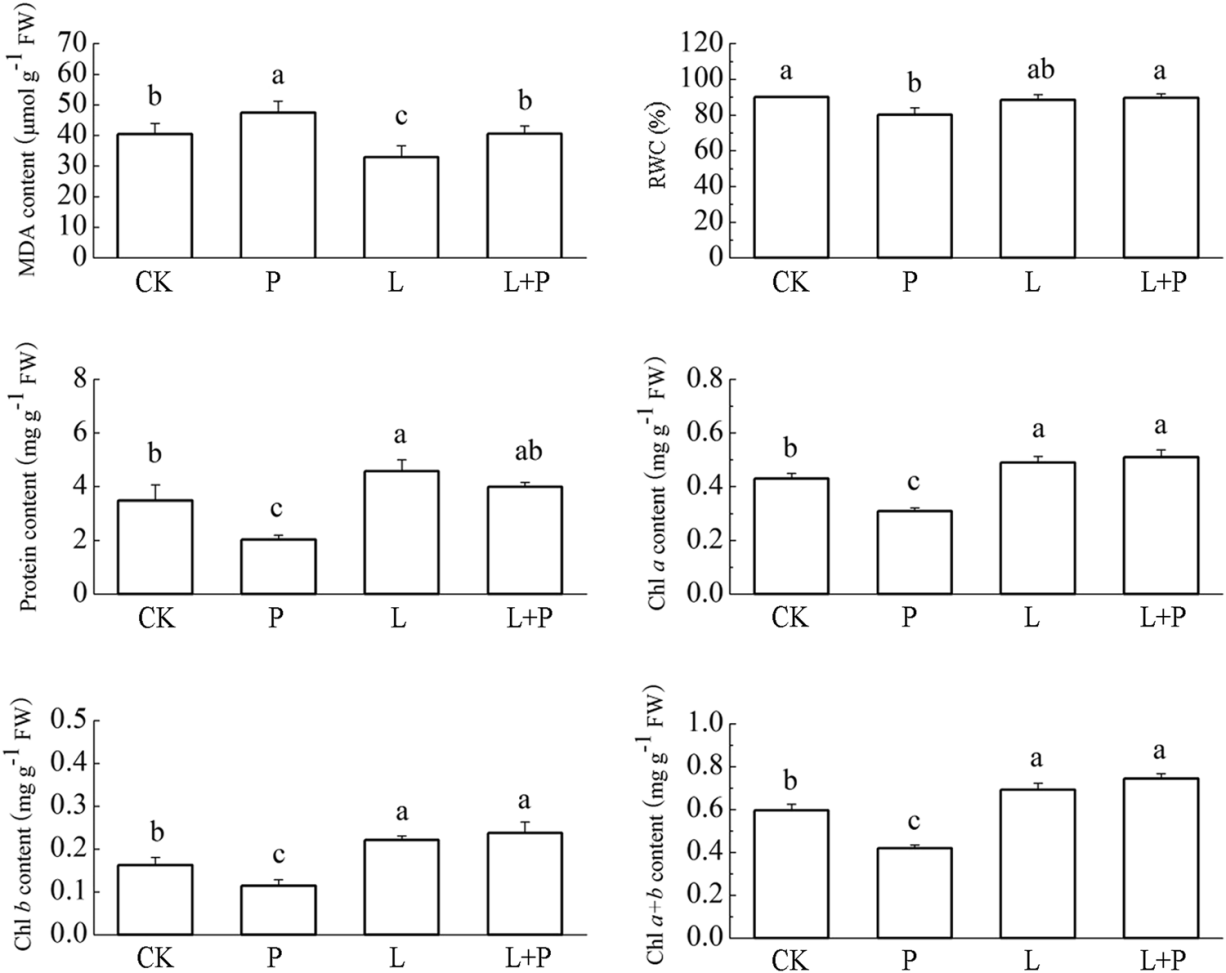
Effect of He-Ne laser pretreatment on the MDA, protein, photosynthetic pigments concentration and the relative water content of the 7-day old seedlings treated with drought stress for 5 days. See notes to Table 1. Bars are means ± standard error of 6 replicates. Different letters above bars indicate significant difference at the 0.05 significant level according to Duncan’s multiple range test.

To assess oxidative stress damage generated by drought stress, MDA content as an important indicator of lipid peroxidation was measured. Drought stress alone for 5 days caused 17.3% increase (p < 0.05) in MDA content compared with the control (Fig. 1). On the contrary, MDA content was decreased significantly (p < 0.05) in wheat seedlings pretreated with He-Ne laser under drought stress compared with drought stress alone. Figure 1 also showed that treatments with He-Ne laser alone dramatically decreased the concentration of MDA compared with the control. These results indicated that He-Ne laser pretreatment could alleviate drought stress induced oxidative damage in wheat seedlings.

As shown in Fig. 1, a remarkable reduction in relative water content (RWC) (12.5%), protein content (71.4%), chlorophyll *a* (38.7%), chlorophyll *b* (42.1%), and chlorophyll (*a* + *b*) (42.2%) content were noticed in drought stress treated wheat seedlings, suggesting that drought stress negatively affected various aspects of wheat seedlings at physiological level. However, He-Ne laser pretreatment combined with drought stress (L + P) significantly improved relative water content, protein and photosynthetic pigments concentration compared to drought stress alone (Fig. 1). Treatments with He-Ne laser alone (L) had no significant effect on relative water content as compared to the control. These results demonstrated that He-Ne laser pretreatment could improve drought stress tolerance in wheat seedlings.

### An overview of transcriptome sequencing datasets

To further elucidate the underlying molecular basis of He-Ne laser irradiation induced drought tolerance, RNA-Seq was employed to investigate the changes of genome-wide gene expression in wheat seedlings pretreated with He-Ne laser irradiation whether or not the seedlings were subjected to drought stress. Approximately 24.96–35.13 million 100 bp paired-end clean reads obtained for the control, drought stress (P), 3 min laser radiation (L) and 3 min laser radiation + drought stress, respectively were generated after adapter trimming and filtering low-quality reads (Table 2). The average Q20, Q30, and GC contents were 95.96%, 89.22%, and 55.11%, respectively and the clean reads of Q20 occupied over than 95% of the total, suggesting high quality sequencing. Then, we used TopHat v2.0.9 software to map all clean reads to the wheat transcriptome. The reads mapping to the reference transcriptome were categorized into two classes: uniquely mapped reads, that are reads that map to only one position in the reference transcriptome, and multi-position match, that are reads mapping to more than one position in the reference transcriptome (Table 2). Of the total clean reads from the four sample groups, 65.82–67.65% were uniquely mapped, and 4.75–6.14% were mapped to multiple loci (Table 2). It has been found that 70.6–73.8% of the reads were mapped to the reference transcriptome. Major mapping reads indicated reliable transcriptome data.

**Table 2:** Summary of RNA-seq data and reads mapping.

Sample name	CK	P	L	L + P
Raw reads	30,890,152	36,407,862	31,799,704	26,286,622
Clean reads	29,987,786	35,133,470	30,566,204	24,9552,36
Clean bases	3.75 G	4.39 G	3.89 G	3.12 G
Q20 (%)	95.15	95.45	95.47	95.3
Q30 (%)	88.82	89.4	89.41	89.12
GC (%)	54.58	55.82	56.15	56.37
Total mapped	19,960,572 (75.56%)	22,695,390 (74.60%)	19,701,309 (74.45%)	15,616,658 (72.58%)
Multiple mapped	2,440,188 (6.14%)	2,442,640 (4.95%)	2,079,018 (4.80%)	1,685,476 (4.75%)
Uniquely mapped	17,220,384 (67.42%)	20,252,750 (67.65%)	17,622,291 (67.65%)	13,931,182 (65.82%)

Different treatment represents: The control (CK), natural drought stress (P), 3 min laser radiation (L), 3 min laser radiation + natural drought stress (L + P).

### Effect of He-Ne laser pretreatment on differentially expressed gene in wheat seedlings under drought stress

**Figure 2:**
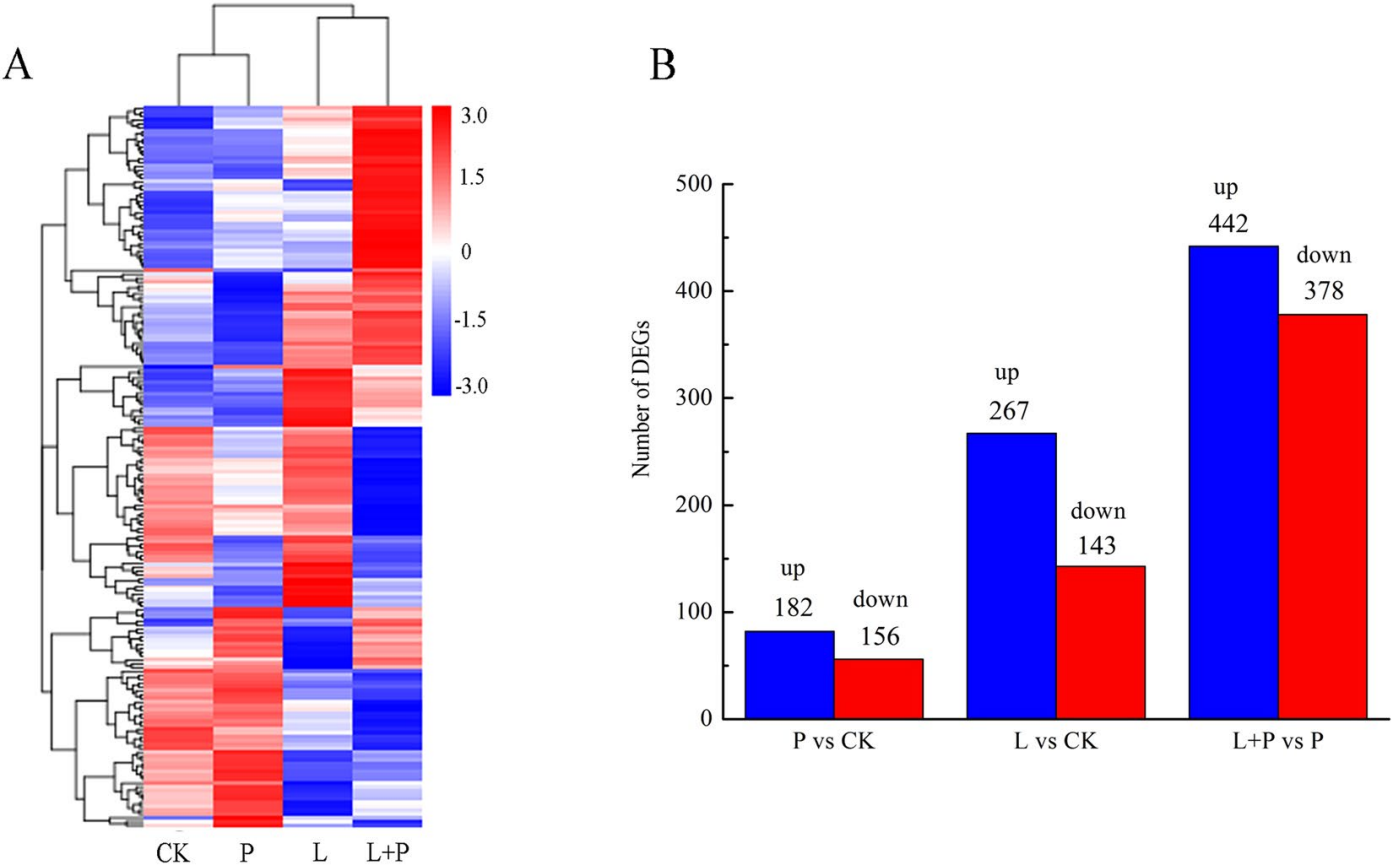
Transcriptome analysis of differentially expressed genes in He-Ne laser pretreated wheat seedlings under drought stress. See notes to Table 1. (**A**) Hierarchical clustering of all the DEGs based on log_10_ RPKM (number of reads per kilobase per million clean reads) values. The color (from red to blue) represents gene expression level from high to low. (**B**) Changes in differentially expressed genes (DEG) in He-Ne laser pretreated wheat seedlings under drought stress. The number of up- and down-regulated genes between P and CK, L and CK, and L + P and P are summarized.

In this study, RNA-Seq analysis was performed to obtain a comprehensive view of gene expression profile in He-Ne laser pretreated wheat seedlings under drought stress. Hierarchical clustering of differentially expressed genes demonstrated that differential gene expression occurred in He-Ne laser pretreated wheat seedlings under drought stress (Fig. 2A). The expression of each gene was normalized to the number of reads per kilobase per million clean reads (RPKM) to compare among different samples. We then used the DESeq R package to identify differentially expressed genes (DEGs). Using false discovery rate (FDR) ≤ 0.05 and fold change ≥1 as the significance cutoff, the expression of 338 DEGs was found to be significantly regulated between drought stress and CK libraries, including 182 and 156 genes that were up- and down-regulated, respectively. The 410 DEGs in both the He-Ne laser alone treatment and CK libraries showed quantitative differences. In addition, we compared L + P and P libraries, and 820 variant genes were found, of which 442 were up-regulated and 378 were down-regulated (Fig. 2B). A Venn diagram showed the distribution of differentially expressed genes from He-Ne laser pretreatment on wheat seedlings under drought stress in Fig. S1. Interestingly, larger numbers of genes were up- or down-regulated at L + P than P, implying that the He-Ne laser pretreatment could induce more complicated transcript regulation in wheat seedlings under drought stress.

### Functional characterization of differentially expressed genes in He-Ne laser pretreated wheat seedlings under drought stress

**Figure 3:**
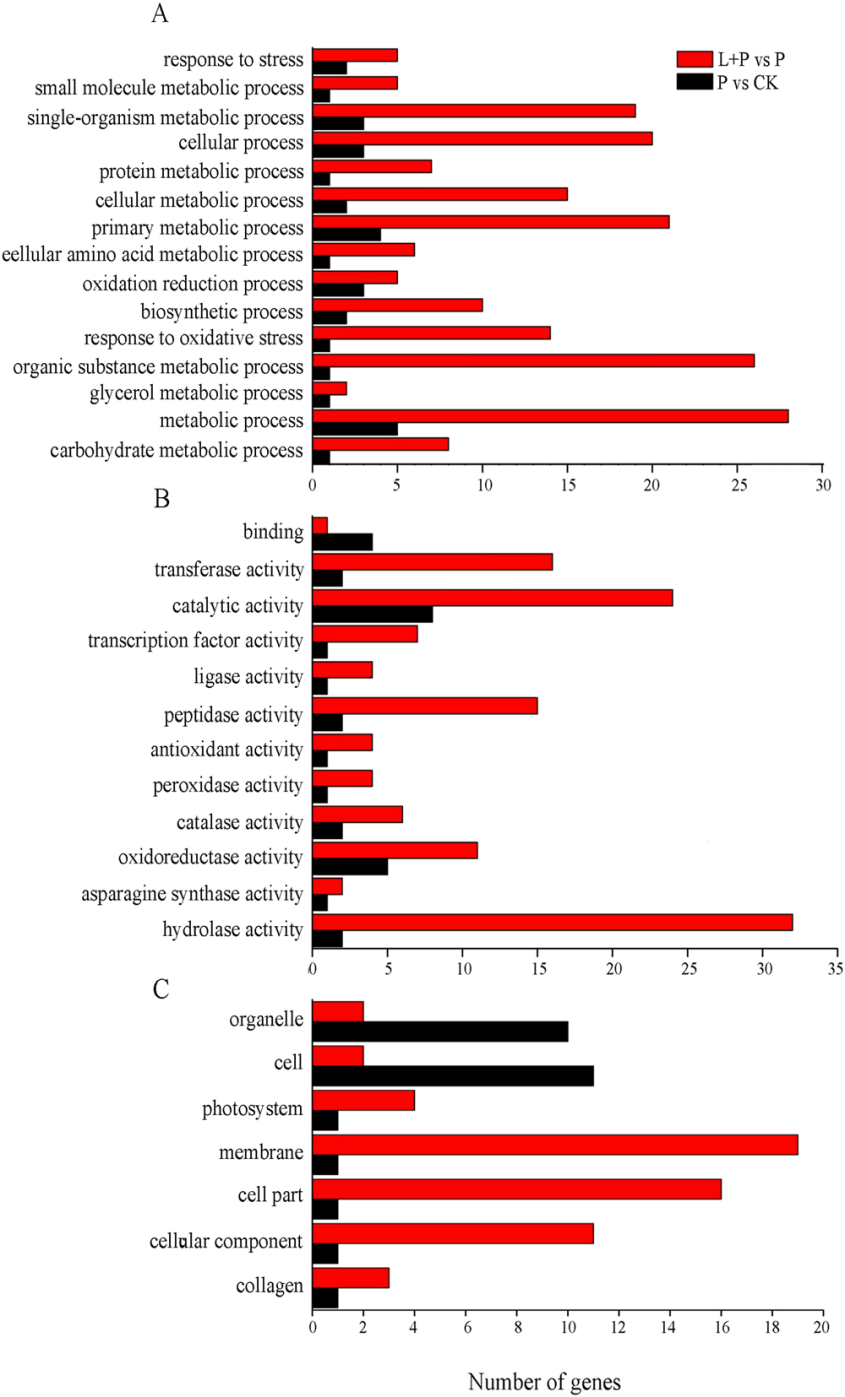
GO functional analysis of differentially expressed genes (DEGs) in P vs CK and L + P vs P. See notes to Table 1. (**A**) Biological process. (**B**) Molecular function. (**C**) Cellular component.

To further investigate the main biological functions of differentially expressed genes in He-Ne laser pretreatment on wheat seedlings under drought stress, gene ontology (GO) analysis were used to classify the functions of the differentially expressed genes. Based on sequence homology, 1,568 DEGs could be categorized into cellular component, molecular function, and biological process, in which there are 7, 12 and 15 functional groups, respectively (Fig. 3). Among these groups, the terms “membrane”, “hydrolase activity”, and “metabolic process” are dominant in each of the three main categories, respectively. Notably, some genes associated with “response to oxidative stress”, “catalytic activity”, “antioxidant activity”, and “oxidation-reduction” were enriched in He-Ne laser pretreatment combined with drought stress, suggesting that He-Ne laser pretreatment induced antioxidant response was activated in wheat seedlings under drought stress.

**Figure 4:**
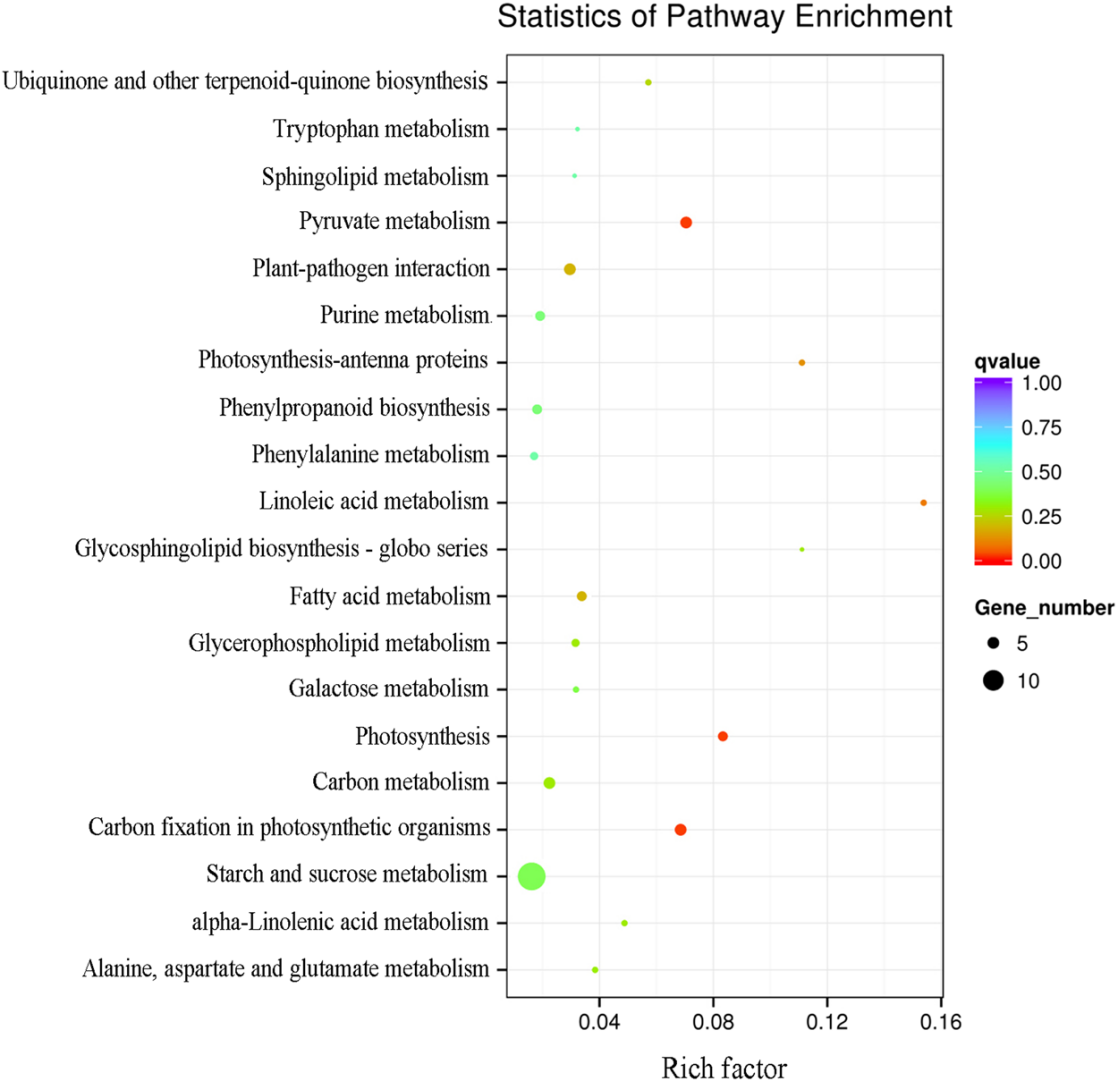
KEGG pathway enrichment analysis of DEGs between P and L + P treatment. The left Y-axis shows the KEGG pathway. The X-axis shows the Rich factor. The enrichment factor indicates the ratio of differential expression unigenes enriched in this pathway to the total number of annotated unigenes. The size and color of each point represents the number of genes enriched in a particular pathway and the *q*-values. A larger enrichment factor value and lower *q*-values indicates a greater degree of enrichment.

To further identify metabolic or signal transduction pathways in which the DEGs are likely to be involved in drought stress tolerance conferred by He-Ne laser irradiation in wheat, pathway enrichment analysis was performed using KEGG database (http://www.genome.ad.jp/kegg/). Among DEGs of He-Ne laser pretreated wheat seedlings combined with drought stress (L + P) and drought stress alone (P) treatments tested the following 6 pathways, with a KEGG pathway annotation, were significantly affected: carbon fixation in photosynthetic organisms, photosynthesis-antenna proteins, plant pathogen interaction, pyruvate metabolism, photosynthesis and linoleic acid metabolism pathways (*q* < 0.05; Fig. 4).

### Differentially expressed genes by He-Ne laser pretreatment involved in drought stress tolerance in wheat seedlings

**Figure 5:**
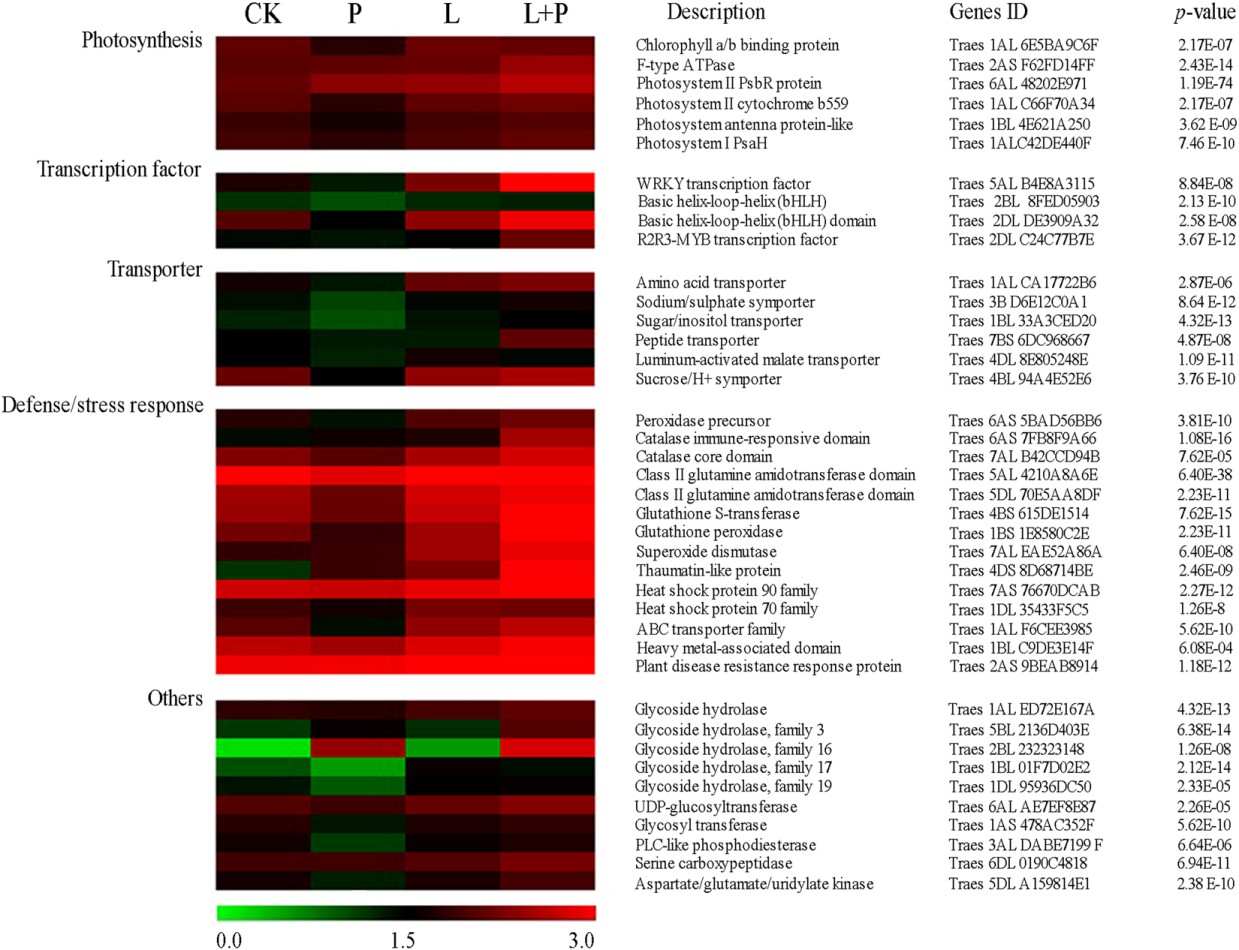
Expression profile of 40 differentially expressed genes involved in transcription regulation, photosynthesis, transport, defense/stress response, and others in He-Ne laser pretreated wheat seedlings under drought stress. The bar represents the scale of the expression levels for each gene (log_10_RPKM (number of reads per kilobase per million clean reads)) in the different treatments as indicated by red/green rectangles. Genes in red indicate up-regulation and in green indicate down-regulation. All genes in this list have a *P*-value for differential expression <10^−5^. The control (CK), natural drought stress (P), 3 min laser radiation (L), 3 min laser radiation + natural drought stress (L + P). Complete information for each gene list can be found in Table S4.

Drought stress changes gene expression in He-Ne laser pretreated wheat seedlings, and several genes expression level exhibited highly dynamic changes (|log_2_Ratio| ≥ 2, Fig. 5), including genes encoding WRKY transcription factors, proteins kinase, stress-associated proteins (plant disease resistance response protein, class III peroxidase, catalase immune-responsive domain, superoxide dismutase, glutathione S-transferase), photosynthesis-associated proteins (photosystem II PsbR protein, chlorophyll a/b binding protein, ATP synthase), transporter-associated proteins (ABC transporter, peptide transporter, drug/metabolite transporter, amino acid transporter, sugar/inositol transporter) (Fig. 5 and Table S4). Interestingly, transcripts of some genes involved in drought stress tolerance accumulated to higher level in He-Ne laser pretreated wheat seedlings than drought stress alone, suggesting that these genes might contribute to the enhanced drought stress tolerance in He-Ne laser pretreated wheat seedlings.

### Quantitative real-time-PCR validation of differentially expressed transcripts from RNA-Seq

**Figure 6:**
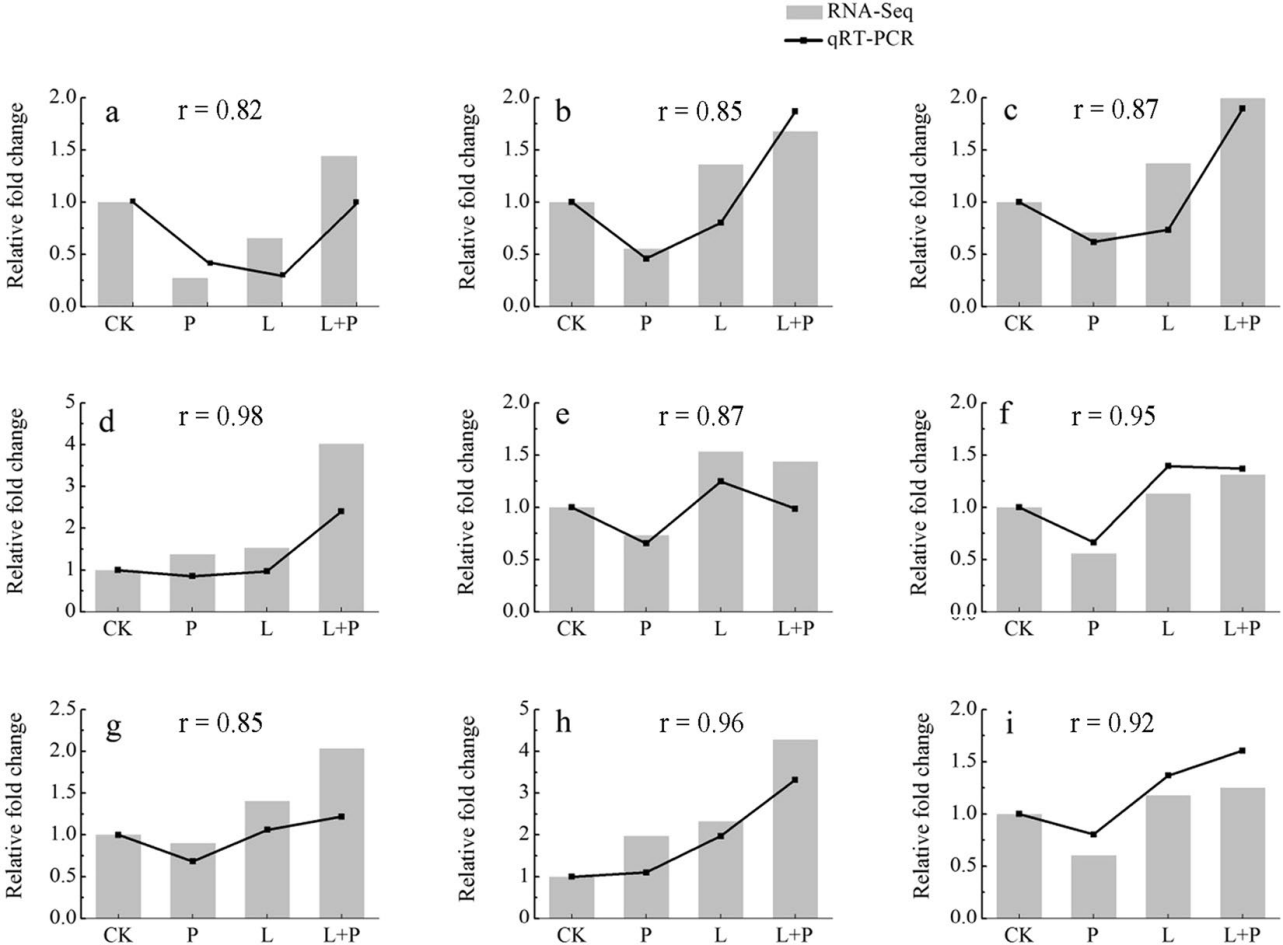
Expression of nine differentially expressed genes in He-Ne laser pretreatment on wheat seedlings under drought stress. Correlation between the expression profiles of the nine transcripts determined by RNA-Seq and qRT-PCR. See notes to Table 1. Genes are (**a**) plant peroxidase; (**b**) class III peroxidase; (**c**) glutathione S-transferase; (**d**) catalase immune-responsive domain; (**e**) heat shock protein 70 family; (**f**) WRKY transcription factor; (**g**) glycoside hydrolase; (**h**) photosystem II PsbR protein; (**i**) drug/metabolite transporter. The y-axes show expression levels (fold difference) determined by qRT-PCR and RNA-Seq.

To further validate the reliability of our Illumina sequencing analyses, we have nine differentially expressed genes by quantitative real time RT-PCR (qRT-PCR) using the independently collected samples that were in different treatments as those used for RNA-Seq, including genes encoding plant peroxidase, class III peroxidase, glutathione S-transferase, catalase immune-responsive domain, heat shock protein 70 family, WRKY transcription factor, glycoside hydrolase, photosystem II PsbR protein, drug/metabolite transporter, respectively. The results presented in Fig. 6 showed that the expression levels of nine genes were higher in He-Ne laser pretreatment combined with drought stress than drought stress alone. The expression trends of these genes agreed with the RNA-Seq data (Fig. 6), and a significant correlation between the expression changes (fold difference) measured by RNA-Seq and those by qRT-PCR (Fig. 6), indicating the reliability of our sequencing data as well as further confirming the He-Ne laser pretreatment conferred improved drought stress tolerance in wheat seedlings.

## Discussion

### He-Ne laser pretreatment on drought tolerance as indicated by morphological and physiological changes

Drought stress adversely influences many physiological processes of wheat, limits wheat growth, and decreases wheat productivity^27, 28^. Results of the current study further confirmed the negative effect of drought stress on plant growth. In this study, our results showed that drought stress significantly inhibited plant height, shoot dry weight, root length and root dry weight, relative water content (RWC), and protein content in wheat seedlings. However, He-Ne laser pretreatment remarkably promoted wheat seedlings growth parameters such as plant height, shoot dry weight, root length and root dry weight, RWC, and protein content under drought stress. The morphological and physiological responses of wheat seedlings suggest that He-Ne laser pretreatment could improve drought stress tolerance in wheat seedlings, in accordance with the enhanced resistance to cold stress^7^, UV-B radiation^12, 13^, cadmium stress^10^, and salt stress^11^. However, the mechanism of how He-Ne laser alleviated the negative effect of drought stress in wheat seedlings need to be further addressed.

### Potential DEGs playing critical roles in drought tolerance conferred by He-Ne laser irradiation in wheat

Drought stress triggers significant molecular and physiological changes in plants, and a global transcriptional reprogramming is considered as the important molecular response of plants to adapt/cope with drought stress. To gain insight into the molecular response of He-Ne laser enhanced drought stress tolerance, we performed RNA-Seq analysis and investigated transcriptional differences in He-Ne laser pretreated wheat seedlings under drought stress. A total of 820 genes (442 up-regulated and 378 down-regulated) were found differentially expressed in He-Ne laser pretreated wheat seedlings under drought stress (L + P) with respective to drought stress alone (P). In addition, we compared P and CK libraries, and 338 variant genes were found, of which 182 were up-regulated and 156 were down-regulated. These results indicated considerable changes of gene expression in He-Ne laser pretreated wheat seedlings under drought stress. Furthermore, functional categories of the DEGs by GO term enrichment analysis in our present study showed that transcripts that function in regulation of photosynthesis and antioxidant defense response were expressed predominantly in He-Ne laser pretreated wheat seedlings. An increasing number of transcriptome studies conducted in diverse plant species have also noted the enrichment of GO terms and metabolic pathways related to antioxidant defense response^29, 30^. Fracasso *et al*. demonstrated that differentially expressed transcripts in photosynthesis, antioxidant and secondary metabolism might play essential roles in tolerant sorghum genotypes adaptation to drought stress^2^. In addition, a large number of genes belonging to various metabolic pathways, such as photosynthesis and carbohydrate metabolism, abiotic stress, regulation of transcription were significantly differentially expressed in He-Ne laser pretreated wheat seedlings, leading us to conclude that most of the genes we identified are involved in response and adaptation to drought stress in wheat.

### He-Ne laser pretreatment enhances photosynthesis by increasing the transcripts of photosynthesis-related genes

Photosynthesis plays a central role as an energy source for plant metabolism^13, 31^. Drought stress inhibits photosynthetic activity in plants by changing chlorophyll content, and a decrease of chlorophyll content implies lowered light harvesting, thus leading to overall growth retardation^27, 31^. In the present study, we found that drought stress significantly decreased chlorophyll content in wheat seedlings, but chlorophyll content was reversed back to a similar level as the control in wheat seedlings pretreated with He-Ne laser. In agreement with these results, Chen *et al*. demonstrated that He-Ne laser treatment of wheat under UV-B radiation significantly increased chlorophyll content and photosynthetic electron transport chain efficiency^13^, thus resulting in the increase of photosynthetic activities. The increased chlorophyll content of wheat seedlings in He-Ne laser pretreatment may reflect changes in expression levels of transcripts regulating photosynthetic activity. Indeed, five genes encoding proteins known to regulate photosynthetic activity were found to be up-regulated in He-Ne laser pretreated wheat seedlings under drought stress. Recently, a number of genes have been reported to be able to regulate drought tolerance through improving the photosynthetic capacity. By using high-throughput sequencing, Zhang *et al*. indicated that an increased expression of a large number of genes related to photosynthesis pathway in upland rice could maintain relatively higher photosynthesis activity under drought conditions^31^, which might result in drought stress adaptation. In this study, He-Ne laser induced the upregulation of most photosynthesis-related genes, involving in PSII PsbR protein, chlorophyll *a*/*b* binding protein and ATP synthase. Furthermore, qRT-PCR from independently generated samples verified that the gene encoding PsbR protein had significantly higher expression in the He-Ne laser pretreated wheat seedlings under drought stress. These results suggest that He-Ne laser irradiation could enhance drought stress tolerance in wheat seedlings by improving photosynthesis at transcriptomic and physiological level.

### Genes encoding transcription factors are important for He-Ne laser pretreated wheat seedlings in response to drought stress

Transcription factors (TFs) are important regulators of gene expression and are known to play crucial roles in various biological processes including responses to drought stress^32–34^. TFs belonging to MYB, AP2/ERF, bHLH, and WRKY families have been well characterized for their regulatory roles in the response of plants to abiotic stress^35, 36^. There have been a number of studies demonstrating that transgenic plants overexpressing genes encoding TFs can greatly enhance their tolerance to various abiotic stresses such as salinity, cold, and drought^37, 38^. The expression patterns of WRKY family were investigated in both the root and leaf of bread wheat under drought conditions. Through *in silico* searches, 35 bread wheat WRKY genes were detected by drought stress and the expression of most of them was known to be down-regulated by drought stress treatment^39^. Furthermore, overexpression of wheat *WRKY* genes could enhance drought tolerance in transgenic tobacco^38^. More recently, several studies also revealed that R2R3-MYB TFs in *Arabidopsis* (*AtMYB44*, *AtMYB60*, and *AtMYB61*) are involved in regulation of stomatal aperture in response to drought stress^40^. Our RNA-Seq data indicated that these genes encoding WRKY, bHLH, and R2R3-MYB TFs were up-regulated in He-Ne laser pretreated wheat seedlings under drought stress, suggesting that these TFs might contribute to the enhanced drought stress tolerance in He-Ne laser pretreated wheat seedlings.

### ROS-scavenging systems are important for He-Ne laser pretreated wheat seedlings in response to drought stress

Drought stress will inevitably result in a massive production of reactive oxygen species (ROS), which can cause lipid peroxidation and leads to oxidative destruction of many cellular structures and components^41–43^. In order to avoid the oxidative damage, plants have developed a complex antioxidative defense system to cope with the stress induced oxidative damages. Many antioxidant enzymes, such as superoxide dismutase (SOD), peroxidases (POD), catalases (CAT) and glutathione S-transferase (GST) play a crucial role in scavenging elevated levels of ROS, thus protecting cells from oxidative damage in plants^21, 42, 43^. It was recently reported that elevated antioxidant levels are associated with tolerance to abiotic stress^44^. Our previous study demonstrated that He-Ne laser pretreatment significantly increased the activities of antioxidant enzymes (SOD, POD, APX and CAT) involved in ROS-scavenging in leaves, and thus enhanced antioxidant ability in wheat seedlings under drought stress^8, 9^. In the present study, the expression of several genes encoding plant peroxidase, class III peroxidase, glutathione S-transferase and catalase were up-regulated in He-Ne laser pretreated wheat seedlings with respect to drought stress alone (Fig. 6). Our results are supported by the results of Fracasso *et al*., who suggested that an increased expression of a large number of genes involved in antioxidant system has been associated with decreased oxidative damage and enhanced stress tolerance in different drought tolerant sorghum genotypes^2^. Using qRT-PCR, Gao *et al*. have also shown that He-Ne laser pretreatment can enhance antioxidant defense systems and improve salt stress tolerance by increasing the gene expression levels of *SOD*, *POD* and *CAT*, which were in accordance with the increase of SOD, POD, and CAT activity^11^. Taken together, our results suggest that the enhanced gene expression of *SOD*, *POD*, *CAT* and *GST* might contribute to the increases in the activities of SOD, POD, CAT and GST in He-Ne laser pretreated wheat seedlings, thus resulting in the protection against oxidative damage induced by drought stress.

### Genes related to solute transport are important for He-Ne laser pretreated wheat seedlings in response to drought stress

Transport processes are very important in the mobilization and accumulation of solutes. Furthermore, these processes play an important role in cell detoxification pathways during adaptation to drought. An increasing number of transporters have been recognized to participate in a multitude of physiological processes that allow the plant to adapt to changing environments and to cope with biotic and abiotic stresses, as well as detoxification processes^45, 46^. Wang *et al*. have demonstrated that several transport-associated unigenes were exclusively detected in *Pyropia haitanensis* in response to rehydration^45^, which encode amino acid transporters, ion transporters, sugar/inositol transporter, and peptide transporter. Many amino acid transporters (AATs) genes are known to function in mitigating water stress conditions in plants, especially by facilitating the transport of stress-related compounds and compatible solutes, such as proline, betaine, and a variety of carbohydrates^46^. Furthermore, over-expression of sucrose transporters (SUTs) has been correlated with improved drought tolerance^47^. Our results indicated that the expression level of genes related to the transport of amino acids and other solutes, including malate, inositol, and sugar were significantly up-regulated in He-Ne laser pretreated wheat seedlings in response to drought stress. The differential expression of these transporters suggested that the uptake and translocation of various nutrients, like sugar, malate, inositol, could be affected by He-Ne laser irradiation. Therefore, it is likely that during water deficit, the upregulation of unigenes encoding various amino acids transporters, peptide transporters, luminum-activated malate transporter and sugar/inositol transporter in He-Ne laser pretreated wheat seedlings could contribute to import of the nutrients and export of secondary metabolites and other toxic compounds that protect wheat seedlings from drought stress.

In conclusion, we analyzed the gene expression profiles of laser irradiation mediated drought-stress tolerance in wheat using RNA-Seq analysis in the present study. Our results showed that 338 genes (182 up-regulated and 156 down-regulated) were differentially expressed in wheat seedlings under drought stress (P) when compared with the control (CK). However, He-Ne laser irradiation induced 820 differently expressed genes, including 442 up-regulated and 378 down-regulated DEGs in wheat seedlings drought stress with respect to drought stress alone, indicating considerable changes of gene expression in He-Ne laser pretreated wheat seedlings under drought stress. Genome-wide transcriptomic analysis presented in this study has expanded our knowledge of this process by identifying significantly altered genes involved in photosynthesis, nutrient uptake and transport, homeostasis control of reactive oxygen species and transcriptional regulation. To the best of our knowledge, this study is the first to characterize potential roles of laser irradiation mediated drought-stress tolerance in wheat at the transcriptional level.

## Electronic supplementary material

Supplementary Information
