# Supplementary material for: Physiological and transcriptome analysis of He-Ne laser pretreated wheat seedlings in response to drought stress
Source: Sci Rep. 2017 Jul 21;7:6108. doi: 10.1038/s41598-017-06518-z (PMC5522386; doi:10.1038/s41598-017-06518-z)
Supplement: Supplementary file 1 — Supplementary Information [file 41598_2017_6518_MOESM1_ESM.pdf]

Physiological and transcriptome analysis of He-Ne laser pretreated wheat seedlings in response to drought stress

Zongbo Qiu*, Mengmeng Yuan, Yanyan He, Yongfang Li, Liang Zhang

College of Life Science, Henan Normal University, Xinxiang 453007, P R China

*Corresponding author:

Zongbo Qiu

College of Life Science, Henan Normal University

Xinxiang, P R China

Phone: +86 373 3326340

E-mail: qiuzongbo@126.com

Table S1 Summary for the wheat transcriptome

Raw Reads	Clean reads	Clean bases	Q20(%)	Q30(%)	GC(%)
47,024,800	45,471,018	6.82G	94.83	87.86	56.69

Table S2 Length distribution of assembled transcripts and unigenes

**Nucleotide length (bp)**	**Transcripts**	**Unigenes**
200-500	98,873	32,288
500-1000	40,452	38,349
1000-2000	33,540	33,483
2000+	15,469	15,468
Total number of reads	188,334	119,588
Minimum length of reads	201	201
Maximum length of reads	17,700	17,700
Average length of reads	801	1,096
Total nucleotide length	150,788,752	131,025,642
N50	1,320	1,520

Table S3 Primer sequences for qRT-PCR

Traes_6BS_0BDACE205 Plant peroxidase<br>F: TGATTCGTCCATCGTCTCG<br>R: CGTGTAGCATTGCCGCTTA
Traes_2DS_708F03DA3 Plant peroxidase<br>F: GCCGTCAACAAGGAGAACC<br>R: ATGCCAGACAGCAGAACAGA
Traes_4BS_615DE1514 Glutathione S-transferase<br>F: GCATCATCATTCCCTTCATC<br>R: GCATCATCATTCCCTTCATC
Traes_6AS_5BAD56BB6 Plant peroxidase<br>F: TTTGCCTCCGACTTCGTG<br>R: TGCAGTTGCGCCTAATCT
Traes_6AS_7FB8F9A66 CAT<br>F: GTCTCAACGTGAAGCCAAGC<br>R: GCACAGTAGGTAATCGACCACA
Traes_5BS_43731B6AC Heat shock protein 70 family<br>F: GTCGTCTAGCAGGAAGCAA<br>R: GGCAAGTCCGTAACAGTGAA
Traes_5BS_43731B6AC Protein kinase domain<br>F: GTGGTGCAGATGCTGGAGGA<br>R: CTGCTGAACATTGAACGGCTAG
Traes_5BL_2136D403E Glycoside hydrolase<br>F: CACCAAAGGGACGAAGCAG<br>R: ACACCAGATCAACCAGCAACA
Traes_6AL_48202E971 Photosystem IIPsbR<br>F: GTTCTGAAACCATCTCCCTC<br>R: GCCTTCTTGCTGGCTACT
Traes_6DL_D582C5427 Drug/metabolite transporter<br>F: TACTGACATGGTCCAACAAA<br>R: AACCCTCCAATAATACTTCC
Tubulin<br>F: GGACCGTACGGGCAGATCT<br>R: CACCAGACTGCCCAAACACA

Table S4 Differential expression genes in He-Ne laser pretreated wheat seedlings under drought stress

Function	Gene ID	P-value	CK	P	L	L+P
**Stress/defense response**						
Plant peroxidase	Traes 6BS_ 0BDACE205	8.42E-17	54.58	14.88	35.83	78.57
Peroxidase precursor	Traes 6AS_ 5BAD56BB6	3.81E-10	43.23	23.86	58.73	72.48
Catalase immune-responsive domain	Traes 6AS_7FB8F9A66	1.08E-16	26.32	36.18	40.29	105.73
Catalase core domain	Traes 7AL_B42CCD94B	7.62E-05	82.60	62.30	107.3	149.20
Class II glutamine amidotransferase domain	Traes 5AL_4210A8A6E	6.40E-38	295.98	163.51	260.76	408.69
Class II glutamine amidotransferase domain	Traes 5DL_70E5AA8DF	2.23E-11	102.36	70.80	146.30	176.55
Glutathione S-transferase	Traes 4BS_615DE1514	7.62E-15	98.60	69.97	135.30	196.74
Glutathione peroxidase	Traes 1BS_1E8580C2E	2.23E-11	75.60	75.20	102.10	16.80
Superoxide dismutase	Traes 7AL_ EAE52A86A	6.40E-08	47.30	50.20	77.20	191.20
Thaumatin-like protein	Traes 4DS_8D68714BE	2.46E-09	15.16	12.62	38.04	38.99
Heat shock protein 90 family	Traes 7AS_76670DCAB	2.27E-12	142.64	136.07	168.73	281.79
Heat shock protein 70 family	Traes 1DL_35433F5C5	1.26E-8	50.88	37.14	78.04	73.08
ABC transporter family protein	Traes 1AL_F6CEE3985	5.62E-10	63.56	25.68	92.35	125.67
Heavy metal-associated domain	Traes 1BL_C9DE3E14F	6.08E-04	123.63	102.52	156.24	184.60
Plant disease resistance response protein	Traes 2AS_9BEAB8914	1.18E-12	178.22	180.0	242.32	379.75
**transcription factor**						
DNA-binding WRKY	Traes 4DL_8AF70D07B	5.38E-10	88.20	91.62	96.20	135.30
WRKY transcription factor	Traes_5AL_B4E8A3115	8.84E-08	95.70	53.52	108.2	125.74
basic helix-loop-helix (bHLH)	Traes_2BL_8FED05903	2.13 E-10	36.50	20.98	42.30	49.32
basic helix-loop-helix (bHLH) domain	Traes_2DL_DE3909A32	2.58 E-08	102.84	86.32	109.54	123.65
R2R3-MYB transcription factor	Traes_2DL_C24C77B7E	3.67 E-12	76.12	60.57	86.32	105.47

**photosynthesis**						
Chlorophyll a/b binding protein domain	Traes 1BE_38BBC0DA1	4.81E-12	185.4	98.45	190.2	216.83
Chlorophyll a/b binding protein	Traes 1AL_6E5BA9C6F	2.17E-07	65.20	22.72	79.50	70.91
F-type ATPase	Traes 2AS_F62FD14FF	2.43E-14	56.20	64.30	71.30	95.42
Photosystem II PsbR protein	Traes 6AL_48202E971	1.19E-74	68.19	134.56	158.18	291.81
Photosystem II cytochrome b559	Traes 1AL_C66F70A34	2.17E-07	56.20	23.50	65.20	87.41
Photosystem antenna protein-like	Traes 1BL_4E621A250	3.62 E-09	30.58	15.70	37.37	52.20
Photosystem I PsaH	Traes 1AL_C42DE440F	7.46 E-10	39.45	23.52	45.67	68.32
**Others**						
Cysteine-rich secretory protein	Traes 7AS_406885D49	2.43E-08	54.84	33.41	1.08	128.90
Glycoside hydrolase	Traes 1AL_ED72E167A	4.32E-13	245.7	95.42	208.35	226.41
Glycoside hydrolase, family 3	Traes 5BL_2136D403E	6.38E-14	62.46	56.06	87.77	127.17
Glycoside hydrolase, family 16	Traes 2BL_232323148	1.26E-08	14.28	36.83	16.64	107.2
Glycoside hydrolase, family 17	Traes 1BL_01F7D02E2	2.12E-14	1.57	250.77	3.82	591.82
Glycoside hydrolase, family 19	Traes 1DL_95936DC50	2.33E-05	0	3.66	37.96	25.56
UDP-glucosyltransferase	Traes 6AL_AE7EF8E87	2.26E-05	23.62	9.00	30.15	37.23
Glycosyl transferase	Traes 1AS_478AC352F	5.62E-10	105.3	76.20	132.1	198.6
PLC-like phosphodiesterase	Traes 3AL_DABE7199F	6.64E-06	58.92	22.20	49.70	63.90
PLC-like phosphodiesterase	Traes 3B_C3C710885	2.87E-06	42.31	13.51	38.20	50.33
Serine carboxypeptidase	Traes 6DL_0190C4818	6.94E-11	79.53	79.26	103.96	164.28
Aspartate/glutamate/uridylate kinase	Traes 5DL_A159814E1	2.38 E-10	44.31	19.76	47.80	16.30
**Transporter**						
Drug/metabolite transporter	Traes 6DL_D582C5427	7.93E-13	236.11	142.91	277.60	295.36
Amino acid transporter	Traes 1AL_CA17722B6	2.87E-06	102.3	56.32	154.24	168.68
Sodium/sulphate symporter	Traes 3B_D6E12C0A1	8.64 E-12	65.42	25.40	78.21	101.48

Sugar/inositol transporter	Traes 1BL_33A3CED20	4.32E-13	45.65	19.62	62.10	89.50
Peptide transporter	Traes 7BS_6DC968667	4.87E-08	89.72	53.27	52.31	147.23
Luminum-activated malate transporter	Traes 4DL_8E805248E	1.09 E-11	91.24	46.93	102.52	79.96
Sucrose/H^+ symporter	Traes 4BL_94A4E52E6	3.76 E-10	154.12	90.99	184.35	208.09

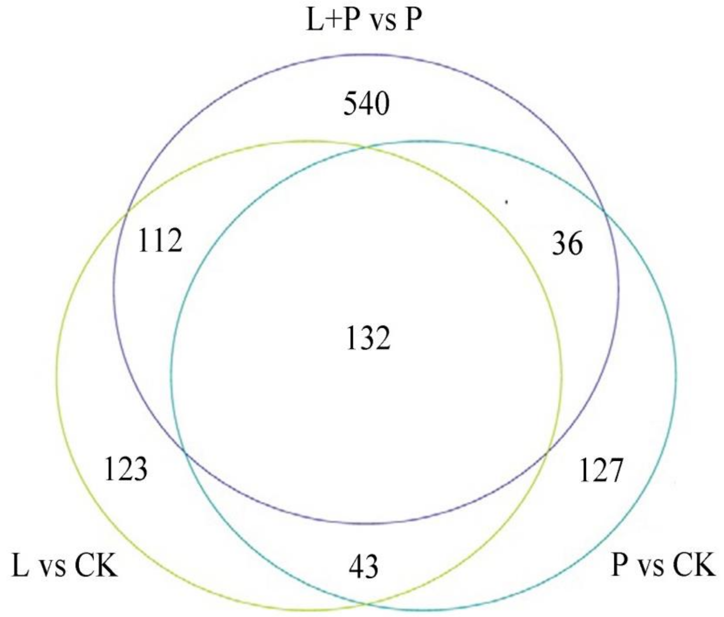

Fig.S1 Venn diagram showing the differentially expressed genes in He-Ne laser pretreated wheat seedlings under drought stress. See notes to Table 1.
